# Meeting unmet needs following minor stroke: the SUN randomised controlled trial protocol

**DOI:** 10.1186/s12913-019-4746-1

**Published:** 2019-11-27

**Authors:** Emma Finch, Michele Foster, Tegan Cruwys, Jennifer Fleming, Philip Aitken, Katherine Jaques, Ian Williams, Darshan Shah

**Affiliations:** 1 School of Health and Rehabilitation Sciences, The University of Queensland, St Lucia, QLD 4072 Australia; 2 Speech Pathology Department, Princess Alexandra Hospital, Brisbane, Australia; 3 Centre for Functioning and Health Research, Metro South Health, Brisbane, Australia; 4 Hopkins Centre, Division of Rehabilitation, Metro South Health, Brisbane, Australia; 5 Menzies Health Institute Queensland, Griffith University, Brisbane, Australia; 6 Research School of Psychology, The Australian National University, Canberra, Australia; 7 Princess Alexandra Hospital, Brisbane, QLD Australia; 8 School of Medicine, The University of Queensland, Brisbane, Australia; 9 Camp Hill Healthcare, Brisbane, QLD Australia

**Keywords:** Minor stroke, Non-disabling stroke, Mild stroke, Unmet needs, Protocol, Intervention

## Abstract

**Background:**

Whilst there are comprehensive guidelines for the rehabilitation of people with severe impairments from stroke, there has been less attention on the health and rehabilitation needs of people with minor stroke. Our study will assess whether a new multi-component service pathway using an integrated model based around primary care will reduce unmet need following minor stroke compared with usual care 1 and 3 months post-hospital discharge.

**Methods:**

One hundred ten patients with minor stroke will be recruited within a parallel, randomised controlled trial design comparing a new service pathway and usual care.

The new service pathway will comprise a self-management kit, customised General Practitioner checklist, and a series of minor stroke educational topics. Participants will complete assessments pre-hospital discharge and 1 and 3 months later. The primary outcome measure will be the Survey of Unmet Needs and Service Usage. Secondary outcome measures will include assessments of ability, adjustment and participation; social group connectedness; return to work; health-related quality of life; and perceptions of the new service pathway (intervention group only). Mixed model repeated measures will be used to analyse within and between group differences at each time point. Return to work will be analysed using Chi square tests. Perceptions of the new service pathway will be analysed qualitatively.

**Dissemination of results:**

The project will produce an evidence-based, multicomponent service pathway for minor stroke patients, applicable to other health services nationally and internationally. Dissemination will include publications and presentations.

**Trial registration:**

Prospectively registered - Australian New Zealand Clinical Trials Registry (ACTRN12619000133134p) 30 January 2019.

## Background

Annually 15 million people worldwide experience a stroke [1]. Medical advances, such as stroke units and hyperacute treatments, have revolutionised acute stroke management. The lessening of stroke severity as a result of these advances has led to an increasing number of strokes classified as “minor”, and more individuals returning to community living post-stroke [2]. Minor stroke is now emerging as a significant health and social issue. Internationally, there are comprehensive guidelines for stroke rehabilitation; however, these guidelines focus on people with more significant impairments post-stroke, with minimal (if at any) information specific to minor stroke (e.g., [3–5]). This is compunded by contention in the literature about the definition of minor stroke [6], and frequent combining of minor stroke with transient ischaemic attack [7, 8].

Minor stroke (also mild stroke or non-disabling stroke) is a stroke where minimal motor deficits and/or no obvious sensory abnormalities are observed in hospital-based assessments [9]. These patients are typically discharged quickly from hospital. They are often left to seek help themselves and receive little ongoing rehabilitation support. Self-managing recovery in the community is challenging, as many patients are ill-equipped to locate services and supports [10] or uncertain about where to get appropriate advice [11]. This is concerning because unmet health, rehabilitation and/or social needs are associated with adverse health consequences, lower life satisfaction and poor psychological wellbeing [12–14]. A systematic review [15] demonstrated that people with minor stroke and their carers experience substantial challenges and disruptions adapting to life post-stroke. Additional research has found that only 69.9% of people with minor stroke return to work 3 months post-stroke [16]. Despite the high incidence of minor stroke in internationally and the personal and societal impact of ongoing limitations in functioning, there is no established pathway for minor stroke management beyond hospital discharge in many countries.

The SUN (Stroke Unmet Needs) study will respond to this problem. The overarching purpose is to compare the effect of a new multi-component service pathway (using an integrated model based on primary care following patients from hospital through to community) for minor stroke on unmet needs at 1- and 3-months post-discharge with patients receiving usual care. The SUN study builds on results of a needs analysis [17, 18] and aims to generate evidence-based recommendations by examining the extent to which the new pathway reduces unmet needs and improves Quality Of Life (QOL) compared to usual care.

### Primary aim

To determine whether a new multi-component service pathway for minor stroke reduces unmet need on the Survey of Unmet Needs (SUNSU) compared with usual care 1- and 3-months post-hospital discharge.

### Secondary aims

1. To assess the effectiveness of the pathway on improving patients’ functional outcomes and QOL as measured by improved scores on the Mayo Portland Adaptability Inventory-4 (MPAI-4), Exeter Identity Transition Scales (EXITS), RAND 36-item Health Survey (SF-36), Stroke Specific Quality of Life Scale (SSQOL), and return to work 1- and 3-months post-hospital discharge compared to usual care.
2. To explore patients’ perceptions of the new pathway.

### Hypothesis

The new pathway will reduce unmet needs at 1 and 3 months and improve functional outcomes, QOL, return to work and service access of minor stroke patients compared to usual care.

Note. Usual care (Control condition): This group will receive the usual care provided to minor stroke patients discharged from the recruiting hospital. This includes provision of an electronic discharge summary to the patient’s General Practitioner (GP) from the hospital treating team and a hospital outpatient follow-up appointment. Requirement for outpatient follow-up is determined at the time of discharge depending on the patient’s medical issues. Additionally, a My Stroke Journey booklet, discharge planning pack, counselling about stroke, medications and follow-up, are often available for patients, although this is not specific to minor stroke. Patients may be referred to private allied health providers or community services at the discretion of the treating team.

## Methods

### Design

In accordance with the Medical Research Council approach [19] for developing complex healthcare interventions, step 1 (needs analysis) [17, 18] has been completed. The current study represents the second step in developing a complex healthcare intervention (Phase II trial). The current project will use a parallel, cross-sectional from hospital to community, randomised controlled trial design (new service pathway compared to usual care) with follow up.

### Study setting

The study will span from a quaternary hospital through to primary care in the community in Queensland, Australia.

### Participants

**Fig. 1:**
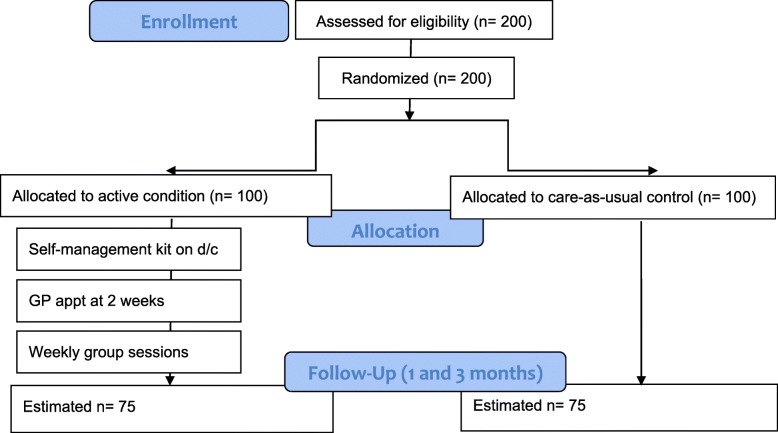
CONSORT diagram of study procedure

One hundred ten participants diagnosed with minor stroke will be recruited while admitted as an inpatient following a minor stroke (See Fig. 1).

### Inclusion and exclusion criteria

Admitted to the recruiting hospital with first ever stroke with minor deficits at the time of discharge (total length of stay in hospital < 2 weeks inclusive of all episodes of care) and having scores on the National Institute of Health Stroke Scale (NIHSS [20], score 0–5) consistent with minor or no obvious deficits. Aged > 18 years (no upper limit) and have no other neurological conditions as documented by the acute stroke team. Patients with a previous history of stroke will be excluded.

### Sample size estimates

For a medium effect size (0.5) with 80% power for the 2 groups, 51 participants are required per group (calculated with G Power*2), increased to 55 to allow for dropout. Our previous project revealed that there were 2979 stroke admissions to the target hospital (Jan 2011-Jan 2017). Approximately 22% of stroke admissions met our inclusion criteria and were referred to our project, with 70% of eligible patients consenting to participate and being retained at follow-up.

### Screening and randomisation

In the 24 h prior to hospital discharge, patients will be screened for eligibility and recruited. The recruitment strategy will involve a two-stream approach over an approximately 6-month period. In the first stream, potential participants will be identified by the stroke team. The second stream will involve pre-screening for potential participants using PowerTrials. Following potential participant identification, a member of the stroke team or a research assistant will approach all eligible people with minor stroke admitted to the recruiting hospital, thus allowing a fair recruitment strategy. Participants will be randomly allocated to receive usual care or the new service pathway. Randomisation will occur using a computerised random number generator (by the primary researcher) with 50:50 assignment to the new pathway or usual care (*N* = 55 participants per group). Assignment will be concealed using opaque envelopes. The participants and their families and the research assistant conducting the assessment sessions will be blinded to the intervention assignment. No circumstances are foreseen which will necessitate unblinding; however, if unblinding is required, the unblinding process for an individual participant will be conducted by the primary researcher.

Participants will consent to participate in the project using a written Participant Information and Consent Form (PICF). PICFs will be provided to potential participants by a member of the stroke team or research assistant. Potential participants will be informed that participation in the trial is entirely voluntary and that they can opt out of the trial.

### Intervention

Intervention: New service pathway: A multi-component pathway comprising 3 parts:

1. Self-management kit: Immediately prior to hospital discharge patients will receive a written information pack from the research assistant (with verbal support at the time). Self-management programs are effective approaches in stroke management [21, 22] and are recommended in the Stroke Foundation Clinical Guidelines for Acute Stroke Management [3]. The kit will include written information about common difficulties specific to minor stroke, the need to see a General Practitioner (GP) after discharge from hospital for ongoing support, need for medications, referral to allied health services, and issues for the patient to discuss with their GP (via checklist). Participants will also receive the My Stroke Journey from the Stroke Foundation, which is a booklet available to all stroke patients in Australia about general stroke information.
2. Customised screening checklist: To encourage patients to undertake self-management in collaboration with their GP, patients will be provided with a checklist to share with their GP. The checklist will include questions about services accessed and residual difficulties/changes post-stroke.
3. Minor stroke sessional educational topics: Patients will be informed about a weekly program of minor stroke community-based educational topics delivered as a group program. These sessions will be facilitated by a qualified health professional and will be scheduled for 1.5 h weekly at rotating locations. Education sessions have been identified as a critical ingredient of successful self-management programs in chronic disease [23]. The group sessions will include brief information about common problems following minor stroke, return to work and driving, need for medications, issues to discuss with GPs, and will have a guest speaker from a different health discipline each time (e.g., physiotherapy, occupational therapy). Information will be “bite sized” (i.e., brief) [24] and patients will receive a short, written summary to take home. The final part of each session will be social, providing patients with a chance to discuss issues, build relationships and receive peer support. To improve adherence/attendance at the education sessions, participants will be sent a text reminder approximately 24 h before each session reminding them of the upcoming session.

The intervention will not be modified for participants in the new service pathway; however, participants will be able to participate in all or only come of the three new service pathway components. Participants’ use of each of the three components will be explored during the study evaluation. Participants in the new service pathway group will be able to receive usual care and referrals to health professionals at the hospital and in the community as individually indicated during the study.

### Outcomes

All participants will complete an initial face-to-face assessment. This will be repeated 1- and 3-months post-discharge. The research assistant administering the assessments will be blinded to participant randomisation group.

#### Primary outcome measure

Survey of Unmet Needs (SUNSU) [13]: Self-rating scale determining needs across impairment, activities of daily living, occupational activities, psychological needs, and community access. The SUNSU has 27-items (e.g., “Travelling in my community”). Participants tick beside an item if they are receiving help with that item and/or if they want help with that item. Each item is scored as “0” if participants do not receive/require help with that item, or “1” if participants require help with that item (therefore scores for each individual item range from “0” no help received or required for that item to “2” help received and required for that item). Higher scores represent more needs. The SUNSU has good person separation reliability and internal consistency scores [13].

#### Secondary outcome measures

1. Mayo-Portland Adaptability Inventory-4 (MPAI-4) [25]: A rating scale completed about ability, adjustment, and participation following acquired brain injury (including stroke). The individual version of the MPAI-4 will be used for the study. Each of the three scales (ability, adjustment, and participation) and the Full Scale MPAI-4 score are converted to T-scores (M = 50, SD = 10) to enable comparison with other adults with acquired brain injury [25]. The MPAI-4 performs well on measures of reliability and validity [25].
2. Exeter Identity Transition Scales (EXITS) [26]: To record social group membership before and after stroke. The EXITS has four subscales to assess social group participation: 1) Group membership before the stroke; 2) Group membership after the stroke; 3) Maintenance of group membership post-stroke; and 4) New group memberships post. For each item, participants respond on a scale from 1 (Not at all true of me) to 7 (Completely true of me).
3. Return to previous occupation (yes/no)
4. RAND 36-Item Health Survey 1.0 (SF-36) [27]: Assesses subjective health and QOL across eight domains: physical functioning, pain, role limitations, emotional well-being, social functioning, energy, and perceptions about general health, and perceived change in health [28]. Items are scored from 0 to 100, with a higher score representing a more favourable state of health. The SF-36 performs well on measures of reliability, central tendency and variability (https://www.rand.org/health/surveys_tools/mos/36-item-short-form/scoring.html).
5. Stroke Specific Quality of Life Scale (SSQOL) [29]: To measure of health-related QOL specific to stroke. The measure consists of 49 items categories into 12 domains: energy, family roles, language, mobility, mood, personality, self-care, social roles, thinking, upper extremity, vision, work and productivity. Participants score each on a score of 1 (“Total help – Couldn’t do it – Strongly agree”) to 5 (“No help needed – No trouble at all – Strongly disagree”) with reference to over the last week. The SSQOL performs well on measures of reliability and validity in mild and moderate stroke patients [29].
6. Semi-structured interview regarding patients’ perceptions of the program: Approximately 25 intervention group patients will be purposively recruited for interview based on key dimensions (e.g. gender, age). Participants will be interviewed by a member of the research team in a mutually agreed location, with interviews lasting no longer than 45 min. Interviews will be audio recorded and transcribed for analysis.
7. *Additional information:* Age, date of stroke, type of stroke, length of stay in hospital, health insurance status, highest level of education, occupation, living situation, and hospital readmissions.

### Data monitoring body

The study will be monitored through regular team meetings and quarterly meetings with the hospital stroke management team. Auditing will occur via the usual HREC auditing process.

### Statistical analysis

Data entry and coding will be undertaken by the research assistant who conducts each assessment session. Missing data will not be entered. Quantitative data will initially be analysed using descriptive analysis (counts, means, standard deviations where appropriate). Mixed model repeated measures will be used to analyse within and between group differences at discharge, and 1 and 3 months on the SUNSU, MPAI, EXITS, SF-36, SSQOL, number of services used, and hospital readmissions. Analyses will occur blinded to group allocation. Subgroup analyses will occur if required. Return to work outcomes will be analysed using Chi square tests. Semi-structured interviews will be analysed using qualitative content analysis [30]. It is not anticipated that interim analyses will occur.

### Ethical and safety considerations

The study has received ethical approval from the Metro South Human Research Ethics Committee (HREC; HREC/2019/QMS/50614). Any changes to the protocol will be submitted to the HREC for review and approval. Once approved, the changes will be disseminated to the research team and relevant other parties. Any adverse events will be reported to the HREC and discussed by the research team.

Potential participants will be informed that participation in the project is voluntary. No payments will be offered to participants. Individuals who decide not to participate in the project will be given usual minor stroke care. Participants will be informed that they are free to withdraw from the study at any point without adverse consequences. Participants will also be informed that their data collected up to the point at which they withdraw from the study will be retained and analysed.

Information obtained in connection with the study that can identify participants will remain confidential. Participants will be given a code instead of their name for labelling paper-based information, demographic information and electronic data files. Paper-based information will be stored in a lockable filling cabinet in the office of the first author. Electronic files will be stored on a password protected computer at the same location. Data will also be stored on The University of Queensland Research Data Manager (RDM) system. Project metadata such as the project name and the collaborators who will have access to the data, will be recorded in a project record within RDM. Within RDM, all project-related data will be stored in a durable format alongside this project metadata, which will be regularly backed up by secure ITS servers. Data will be accessible only to the collaborators recorded on the project record, and will be only accessible via their institutional usernames and passwords. All data will be stored for 7 years.

If participants report ongoing stroke-related issues in their final assessment session, the research assistant will provide written information for participants to take to their GP rquesting a referral to the appropriate health professional for foolw up care.

### Expected outcomes

The project outcome will be an evidence based, multicomponent service pathway for minor stroke. The pathway could be utilised within other health services throughout Australia and internationally. Research translation will occur via “actionable nuggets” [24] in which key information from the project is disseminated to relevant groups as “bite sized” information on postcards (including GPs, hospital staff). It is expected that the project results will be disseminated via 1–2 publications in international, peer-reviewed journals and be presented at 2 national/international stroke conferences. It is anticipated that the publications arising from the study will be written by the primary research with input from the research team. It is anticipated that the research team will meet authorship eligibility criteria. No professional writers will be used. At the completion of the study, participants will be directed towards any publications that have arisen from the research.

## Discussion

Minor stroke is a growing health issue worldwide. In minor stroke, post-stroke disabilities may not become apparent until patients have returned home and attempted to resume their previous activities (e.g., work, household responsibilities). This population may also struggle with recovery due to poor awareness of local service availability. There is therefore an urgent need to develop a new service pathway to meet the needs of minor stroke patients in order to reduce the burden of disease. Our study will meet this need by comparing a new service pathway with usual care following minor stroke in a Phase II trial. The results of the study will likely lead to a change in practice, and assist is providing coordinated hospital to community services for this under-served patient population.

## Data Availability

Study data and materials will be stored and available on the University of Queensland RDM system. The datasets used and/or analysed during the study will. be available from the corresponding author on reasonable request. No confidential patient data will be shared.

## Abbreviations

EXITS: Exeter Transition and Identity Scales
GP: General Practitioner
HREC: Human Research Ethics Committee
MPAI-4: Mayo Portland Adaptability Index-4
NIHSS: National Institutes of Health Stroke Scale
PICF: Participant Information and Consent Form
QOL: Quality of Life
RDM: Research Data Manager
SF-36: RAND 36-Item Health Survey
SSQOL: Stroke Specific Quality of Life Scale
SUN: Stroke Unmet Needs
SUNSU: Survey of Unmet Needs
